# From Speed and Sensitivity to Robust Reliability: Addressing the Detection Bias of Loop-Mediated Isothermal Amplification (LAMP) in Food Authentication

**DOI:** 10.3390/foods15193483

**Published:** 2026-09-29

**Authors:** Xiong Xiong, Yahui Zhang, Mengxuan Liu, Yan Guo, Ying Yang

**Affiliations:** 1 College of Food Science and Light Industry, Nanjing Tech University, Nanjing 211816, China; 2 School of Horticulture, Anhui Agricultural University, Hefei 230036, China

**Keywords:** food adulteration, loop-mediated isothermal amplification, on-site detection, analytical reliability, operational robustness, visual detection

## Abstract

Food adulteration poses severe threats to the integrity and safety of global food supply chains. Innovations in analytical techniques, especially the rapid development of LAMP-based detection methods, are indispensable for addressing these risks. However, the dominant research paradigm, which has long prioritized accelerated amplification kinetics and ultralow detection limits, largely obscures a critical limitation: insufficient detection reliability when deployed in complex field environments. This review consolidates state-of-the-art advances targeting reliability optimization and delineates key technical strategies to construct robust, improved-reliability LAMP platforms for real-food testing. Specifically, we summarize critical factors at the reaction and readout levels that compromise the reliability of LAMP under realistic scenarios, such as inexperienced operators, degraded samples, matrices containing inhibitory substances, and uncontrolled environments. We further elaborate on effective solutions to bridge the reliability gap between laboratory validation and on-site deployment, which requires coordinated technical improvements throughout the full LAMP testing pipeline. Ultimately, we propose a forward-looking developmental principle for next-generation LAMP assays: analytical reliability and operational robustness should be elevated to equal priority alongside the traditionally pursued ultra-rapid amplification and high sensitivity. This balanced technical framework may help pave the way toward large-scale standardization of LAMP-enabled food authentication, although its presumptive results must be confirmed by an independent validated method before they can support regulatory enforcement or other formal decisions.

## 1. Introduction

Food safety has emerged as a paramount global public health priority due to its profound impacts on human wellness and the sustainable development of the food sector. Despite remarkable improvements to food regulatory frameworks globally, food safety assurance remains persistently challenged by adulteration, as exemplified by several well-documented incidents, such as Europe’s horse-meat fraud scandal and China’s melamine-tainted milk crisis [1,2]. Recent investigations reveal that strong market demand and substantial profit margins make high-value edible oils, especially camellia oil and olive oil, attractive targets for fraudulent dilution and mislabeling [3]. The annual seafood expenditure of Spanish households affected by such fraudulent activities reached €683.3 million [4]. Widespread malpractice also includes blending brick dust into red chili powder, a dangerous adulterant that inflicts irreversible chronic organ damage on consumers upon long-term ingestion [5]. Beyond this case, food adulteration could trigger a broader spectrum of adverse health outcomes, including acute methanol poisoning from tainted alcohol, long-term carcinogenic risks from aflatoxin-contaminated nuts, and severe allergic reactions caused by undeclared allergens in falsely labeled allergen-free food [6,7].

Analytical techniques are essential for food authentication and adulteration detection in the current regulatory and safety frameworks. Over the past two decades, the analytical toolkit has expanded dramatically, among which nucleic acid-based techniques have attracted increasing attention [8]. This trend stems mainly from DNA’s favorable chemical stability against common food-processing stresses such as heat, high pressure, and chemical treatments [1]. These properties make nucleic acids suitable biomarkers for detecting adulterants in complex processed-food matrices. Particularly, isothermal amplification methods have emerged as powerful alternatives to conventional thermal cycling-based nucleic acid detection (Table 1). By eliminating the need for intricate thermal regulation systems, these methods enable streamlined operational workflows and markedly accelerated reaction kinetics [9]. Widely adopted isothermal techniques, including loop-mediated isothermal amplification (LAMP), recombinase polymerase amplification (RPA), rolling circle amplification (RCA), and strand exchange amplification (SEA), have been systematically reviewed elsewhere [10]. In particular, as first reported by Notomi et al. [11], LAMP stands out as a representative isothermal nucleic acid amplification technique that employs four to six primers to achieve rapid, highly specific amplification under constant-temperature conditions (typically 60–65 °C). This method is capable of amplifying DNA templates to yield up to 10^9^ copies within 60 min, generating unique stem–loop DNA amplicons with variable stem lengths.

Recent years have seen a surging development of LAMP-based analytical strategies for food adulteration identification [24]. Notably, validated LAMP assays have achieved a limit-of-detection (LOD) as low as 0.1% (*w*/*w*) for the detection of genetically modified samples (maize or soybean) [25]. Further optimized LAMP protocols targeting porcine mitochondrial DNA are capable of identifying pork adulteration at a concentration of merely 0.05% (*w*/*w*) within thermally processed beef meatballs [26], while LAMP assays for high-value goat milk adulteration reach a LOD of 5% (*v*/*v*), both outperforming conventional PCR in sensitivity [27]. Beyond superior sensitivity, the streamlined LAMP workflow supports rapid on-site implementation. Integrated LAMP systems paired with rapid crude DNA extraction can finish on-site screening of raw meat samples at processing lines within 40 min [28]. Meanwhile, the CRISPR/Cas12a integrated LAMP assay offers reliable and accurate results for silkworm identification in 30 min [19]. Moreover, lateral flow strip LAMP platforms complete multi-component adulteration identification for port customs clearance in less than 20 min, satisfying the stringent requirements for rapid field-based food safety surveillance [29].

Moreover, recent innovative advances have further refined LAMP performance via integration with novel signal transduction platforms to alleviate intrinsic technical limitations [30]. For instance, CRISPR-coupled LAMP systems stand out as a powerful strategy to tackle non-specific amplification, the primary source of LAMP-induced false positives [31]. Compared with conventional standalone LAMP assays, a one-tube LAMP-CRISPR/LbCas12a POCT platform has achieved around 10-fold improved analytical sensitivity [18]. More critically, thermophilic Cas12b variants have recently been engineered to function at 62 °C, supporting fully closed-tube, single-pot reactions [32]. These breakthroughs overcome the long-standing temperature incompatibility bottleneck and, more importantly, eliminate aerosol contamination hazards endemic to two-step open-tube operating protocols. Complementing advances in target specificity, digital LAMP (dLAMP) enables the transition of LAMP from qualitative screening to absolute quantification—a capability of critical importance for regulatory enforcement, where legal and economic ramifications hinge on statutory adulteration threshold limits [33]. Electrochemical sensing represents another promising readout route that circumvents bulky optical hardware such as UV light sources and fluorescence detectors [34]. Furthermore, functional nucleic acids have also been advocated to boost sensitivity and point-of-care compatibility for next-generation LAMP [35]. Finally, when paired with artificial intelligence (AI)-driven automated signal decoding to eliminate subjective human bias during endpoint assessment, such integrated platforms lift LAMP’s overall reliability to a standard comparable to qPCR, the prevailing gold-standard molecular detection technique [36].

Despite these analytical strengths in LAMP-based food authentication, the practical reliability of LAMP testing remains a severely under-investigated critical issue. Although multi-mode readout platforms have expanded LAMP versatility [37], regulators still lack sufficient standardized experimental evidence to judge whether a single presumptive positive LAMP result justifies mandatory food recall operations. This lack of industry consensus arises not from regulatory reluctance, but from a systemic research gap in food-authenticity research. Specifically, the dominant research paradigm, which prioritizes accelerated amplification kinetics and ultralow detection limits, systematically obscures a critical limitation about the reliability deficit of standard LAMP assays when deployed in field conditions. Their performance is highly vulnerable to multiple confounding factors at both reaction and readout stages.

Recent excellent review articles have summarized LAMP chemistry, indicator systems, and broad food-related application landscapes for both pathogen monitoring and food authentication [9,24,30,35]. Nevertheless, most existing reviews primarily catalog assay performance, detection-limit metrics, and available readout modalities. Reliability loss upon transition from well-controlled laboratory settings to complex real-world food-surveillance environments is frequently mentioned only briefly as a secondary limitation, rather than treated as the central organizing theme of the review. Against this background, the present narrative critical review has three explicit objectives: (i) we systematically categorize and analyze reaction-level and readout-level factors responsible for LAMP performance deterioration in field food-authentication testing; (ii) we synthesize a coordinated five-dimensional optimization framework linking documented practical vulnerabilities with corresponding technical countermeasures; and (iii) we advocate a paradigm shift in LAMP assay development, where analytical reliability and operational robustness are prioritized on an equal footing alongside amplification speed and ultralow detection limits. This work further highlights gaps between laboratory-validated LAMP assays and the evidentiary expectations that govern regulatory-relevant food-authenticity surveillance.

## 2. Review Methodology

Scholarly publications reviewed in this paper were identified through searches of journal databases and subject-specific professional websites (Google Scholar, PubMed and Web of Science). Search terms including LAMP, food authentication/adulteration, field-test reliability, matrix inhibition, lyophilized reagents, internal amplification control, digital readout, and CRISPR-coupled LAMP were utilized. Preliminary relevance screening was performed based on article titles and abstracts. Full-text evaluation was conducted for records passing the initial screening step. To expand the literature coverage and capture recent advances, reference lists of highly relevant papers and the “cited-by” function in Google Scholar were employed as a snowballing strategy to identify additional eligible publications.

The inclusion criterion for selected papers requires that they be peer-reviewed and directly relevant to the topic of LAMP-based analytical performance, reliability bottlenecks, technical optimization, or field deployment for food authenticity. Only journal articles written in English from 2020 to date were considered for the review. This narrative synthesis prioritizes conceptual synthesis and critical interpretation of technical routes rather than exhaustive quantitative meta-analysis. Therefore, potential literature-selection bias cannot be fully excluded. Selected representative studies are summarized in Table 1.

Finally, for clarity, key terms used throughout this review are explicitly defined below: food authentication: the analytical process of verifying whether food matches its declared identity, species, geographic origin or labeling composition. This represents the primary scope of the present review. Food adulteration: a major subset of food fraud, referring to intentional, economically motivated substitution, dilution or undeclared addition of ingredients into food products. Food fraud: an umbrella term covering food adulteration, mislabeling, counterfeiting and other intentional deceptive practices in food supply chains. Reliability: the overall capacity of a LAMP assay to deliver consistent, defensible test outcomes under real-world on-site food-authentication conditions, beyond well-controlled ideal laboratory environments. A positive LAMP result is a presumptive indication of possible non-compliance, not a finding of non-compliance. Conversely, a negative LAMP result only supports the absence of the target above the validated limit-of-detection and cannot exclude adulteration below that level.

## 3. The Gap—Where Reliability Crumbles

**Figure 1:**
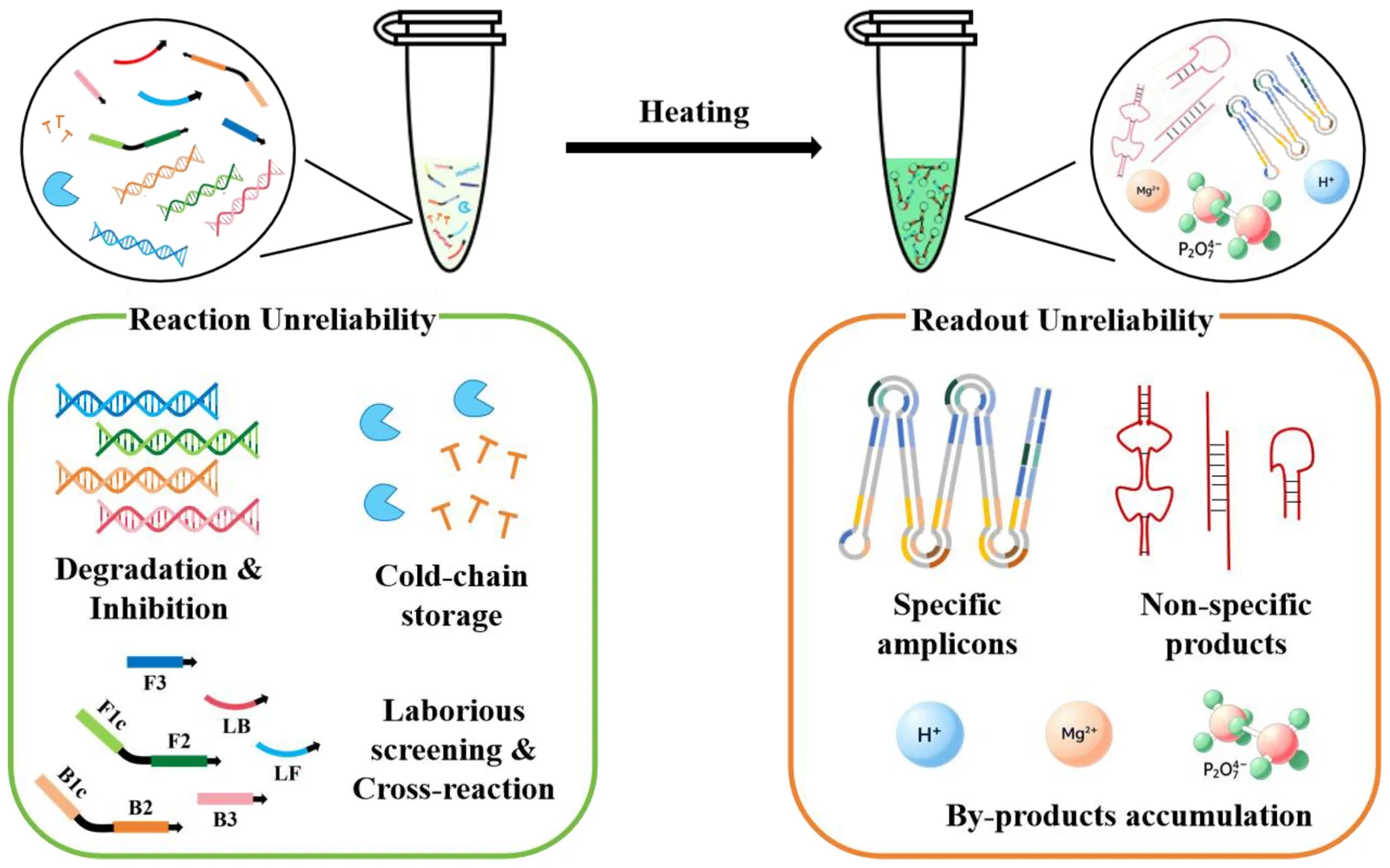
Schematic illustration of two distinct layers of unreliability limiting LAMP performance. The whole LAMP workflow generally suffers from two sequential risk clusters. Reaction unreliability occurs prior to isothermal heating, involving template degradation, sample inhibition, strict cold-chain storage, and complicated primer design with risks of cross-reaction. Readout unreliability emerges after amplification, driven by the simultaneous generation of specific amplicons and off-target products, coupled with the accumulation of inorganic byproducts that interfere with signal interpretation.

Over the past two decades, LAMP has proven itself one of the most robust molecular tools for food adulteration detection [24]. Despite the excellent analytical performance under well-controlled laboratory conditions, LAMP suffers drastic deterioration in reliability when transferred to field on-site surveillance, driven by a wide array of interfering variables that have yet to be quantitatively characterized in current research. As illustrated in Figure 1, the compromised field reliability can be categorized into two core defect clusters: unreliable amplification reactions and unreliable endpoint signal interpretations. Specifically, reaction-level unreliability mainly stems from three inherent systemic flaws of LAMP reaction systems: compromised integrity and inferior quality of template DNA extracted from intricate food matrices, suppressed amplification efficiency arising from suboptimal primer design and uncalibrated reaction parameters, and limited cold-chain-free stability of liquid-phase reaction reagents. By contrast, readout-associated unreliability primarily stems from inherent weaknesses of traditional visual endpoint detection: high subjectivity of naked-eye discrimination, lack of multiplexing capacity for simultaneous multi-target identification, frequent false-positive signals triggered by off-target amplicons, and elevated aerosol cross-contamination risks upon post-amplification tube opening. These two categories of unreliability are not merely technical drawbacks, but substantial vulnerabilities bearing regulatory and public health repercussions with divergent risk profiles. For instance, false-negative results allow adulterated food commodities to reach consumers [38], while false-positive results impose unnecessary losses on compliant food manufacturers [39].

Importantly, for the moment, LAMP is best conceptualized as a preliminary rapid screening tool. Any positive, trace-level or borderline-weak-positive LAMP screening result cannot constitute standalone evidence for regulatory enforcement or formal administrative decisions. Suspect screening outputs must undergo replicate testing, evaluation of internal-amplification-control status, and orthogonal accredited confirmatory analysis before regulatory or legal conclusions can be drawn. This screening-then-confirmation paradigm aligns with internationally accepted principles for official-control molecular testing described in ISO/TS 16393:2019 [40] and EU analytical-control guidance for food matrices. Until these bottlenecks are systematically characterized and adequately addressed, LAMP will remain a practical preliminary screening tool rather than a credible, regulatory-compliant analytical instrument.

### 3.1. Reaction Process Unreliability

#### 3.1.1. Sample Quality and Matrix Inhibitor Interference

LAMP is widely recognized for superior anti-inhibition capacity relative to conventional PCR, a trait largely attributed to the robust strand-displacement activity of *Bst* DNA polymerase and multi-primer recognition architecture [41]. Moderate levels of co-extracted contaminants barely suppress amplicon accumulation or disrupt stem–loop product structures, enabling stable turbidity and colorimetric readouts even from minimally purified crude DNA templates [14]. Nevertheless, such anti-interference capability is conditional rather than absolute, mandating rigorous empirical validation for unprocessed, field-collected food matrices without purification pretreatment.

Suboptimal nucleic acid extraction pipelines are a primary driver of compromised LAMP performance, mainly stemming from incomplete cellular lysis, endogenous nuclease-mediated template degradation, and co-elution of matrix-derived inhibitory compounds that collectively retard amplification kinetics and lower overall detection accuracy [42]. Seven canonical inhibitory substances ubiquitous in biological and food matrices—bile salts, calcium chloride, hematin, humic acid, immunoglobulin G, tannic acid, and urea—exhibit concentration-dependent suppression of LAMP strand-displacement activity [43]. At threshold concentrations comparable to those that fully abrogate PCR, these compounds delay exponential amplification onset and quench fluorescent reporter signals, generating ambiguous weak-positive outputs prone to misjudgment.

In food systems, inhibitor-related reliability risks are drastically amplified by the extreme chemical diversity of commodity food matrices, which introduce unique inhibitory mechanisms rarely observed in clinical specimens. For instance, starch granules and chitosan polysaccharides abundant in cereals, aquatic products and agricultural crops form viscous microenvironments that physically block primer–template hybridization and hinder *Bst* DNA polymerase catalytic turnover, slowing or completely halting amplification progression [32]. Polyphenols and condensed tannins enriched in fruits, vegetables and tea leaves exert irreversible dual inhibitory effects. These aromatic metabolites tightly bind both negatively charged genomic DNA and the catalytic domain of strand-displacing polymerase, triggering permanent reaction suppression that cannot be rescued solely via buffer formulation adjustment [44]. Furthermore, high residual salinity, food-processing-derived surfactants and acidic metabolic byproducts disrupt the tightly buffered ionic strength and pH homeostasis required for optimal LAMP enzyme activity, further delaying amplification initiation and reducing total amplicon yield [45]. While LAMP tolerates low-to-moderate levels of food-derived inhibitors more effectively than PCR, high concentrations of these matrix contaminants can completely abrogate amplification, limiting the direct detection of unprocessed high-viscosity and polyphenol-rich food samples [46]. Additionally, such matrix interference also disproportionately impairs visual readout interpretation, as faint amplicon signals are masked by pigmented food extracts even when target amplification partially proceeds [9].

Compounding this matrix interference risk is the largely understudied impact of template DNA degradation on LAMP diagnostic performance [47]. Unlike conventional qPCR, which targets short 100–300 bp amplicons, canonical LAMP assays typically amplify 200–400 bp conserved mitochondrial or nuclear gene fragments, requiring six spatially distinct primer-binding loci to remain fully intact for successful exponential amplification [48]. When food specimens undergo prolonged ambient storage, repeated freeze–thaw cycles, or high-temperature pasteurization, random nuclease cleavage and chemical depurination generate fragmented DNA with average fragment lengths below 200 bp [49]. This partial degradation does not fully eliminate target nucleic acid, but it statistically disrupts the spatial arrangement of LAMP primer-binding sites, generating characteristic delayed amplification or ambiguous weak-positive signals that cannot be readily distinguished from partial matrix inhibition. This dual confounding effect accounts for a large proportion of inconsistent LAMP results between fresh reference standards and field-stored commercial food specimens reported in recent surveillance studies. Collectively, these findings underscore that standardized sample preservation protocols and streamlined matrix-adapted pretreatment pipelines are indispensable to guarantee consistent, replicable, and trustworthy LAMP detection for field food adulteration screening.

#### 3.1.2. Primer Design Barriers and Optimization Bottlenecks

Unlike conventional PCR that utilizes only a single primer pair, canonical LAMP assays require four to six oligonucleotides that collectively recognize six to eight discrete conserved regions of the target nucleic acid [50]. This elaborate multi-primer architecture underpins LAMP’s exceptional amplification efficiency and intrinsic specificity, yet it simultaneously introduces inherent structural vulnerabilities and labor-intensive developmental bottlenecks that severely undermine assay reproducibility in field applications.

First, current LAMP primer design workflows heavily rely on bioinformatic platforms, such as PrimerExplorer, NEB LAMP Primer Design Tool, and LAMPrimers iQ, which predict melting-temperature (Tm) profiles and screen potential intramolecular secondary structures in silico. However, computational simulation cannot fully recapitulate the complex biochemical microenvironment of crude food matrix amplification, frequently leading to poor consistency between predicted primer feasibility and actual experimental performance [51]. In practical assay development, LAMP primer design and optimization remain highly empirical and unstandardized. The absence of unified thermodynamic screening benchmarks, cross-reactivity cutoff thresholds, and quantitative robustness evaluation criteria reduces the entire design pipeline to an opaque trial-and-error process, accompanied by unpredictable background interference and amplification bias [48]. As a result, each candidate primer set necessitates extensive iterative optimization, including primer sequence replacement, concentration gradient tuning, buffer component adjustment, and temperature calibration [52,53]. Such repeated trial-and-error validation imposes considerable labor and time costs, making LAMP primer development far less streamlined, low-throughput, and less standardized than traditional PCR primer preparation.

Second, the coexistence of multiple oligonucleotides and ultra-long inner primers constitutes an inherent structural fragility that destabilizes LAMP reaction systems and triggers pervasive non-specific amplification (NSA) artifacts [54]. Particularly, the core functional inner primers (FIP and BIP) are 40–55 bases in length, vastly longer than conventional PCR primers, which renders them thermodynamically susceptible to stable hairpin self-folding [55]. Complex intermolecular interactions—including spontaneous homodimerization, heterodimer formation and off-target cross-priming—represent the dominant failure modes that degrade assay specificity and run-to-run reproducibility [56]. Compounding this limitation, LAMP requires substantially higher overall primer concentrations to sustain robust strand-displacement activity, which exponentially elevates the probability of unplanned primer–primer hybridization [57]. These spontaneous primer–primer interactions can trigger template-independent aberrant amplification, generating persistent background noise and recurrent false-positive signals that invalidate quantitative and qualitative field testing results [58].

In recent years, a suite of novel strategies have been proposed to mitigate primer-triggered NSA and dimer artifacts. For instance, simplified short-length LAMP variants with truncated primers reduce oligonucleotide cross-complementarity and suppress template-independent false amplification [59]. Post-amplification high-resolution melting analysis, coupled with saturated fluorescent dyes, enables clear discrimination between genuine target amplicons and short primer-dimer byproducts, offering a straightforward post hoc route to eliminate false-positive misclassification [60]. Additionally, blocker oligonucleotides targeting the F1c/B1c domains of inner primers effectively suppress off-target amplification, shorten time-to-positive values and lower overall false-positive frequencies [61]. Despite these promising auxiliary approaches, such remedial tactics only alleviate superficial interference rather than fundamentally resolve the intrinsic structural defects embedded within multi-primer LAMP architectures. Therefore, the labor-intensive multi-round optimization workflow and the intrinsic biochemical instability of long-sequence multi-primer systems constitute the two dominant bottlenecks that restrict the standardization, repeatability, and large-scale field application of LAMP detection technology.

#### 3.1.3. Reaction Optimization and Reagent Stability

The amplification efficiency, specificity, and reproducibility of LAMP assays are highly dependent on the precise fine-tuning of intrinsic reaction parameters. Among all adjustable variables, incubation temperature, magnesium ion dosage, primer concentration gradients, and dNTP loading represent four core tuning factors that jointly shape reaction performance [62]. Subtle deviations can dramatically alter amplification kinetics, narrow detection windows, and introduce pronounced run-to-run variability—an inherent barrier to standardized field-level deployment of LAMP food authentication.

LAMP is inherently constrained by a narrow thermal window of 60–65 °C, a threshold required to simultaneously sustain robust *Bst*-mediated strand displacement and stringent specific primer annealing [11]. Rigorous temperature calibration therefore becomes an indispensable developmental step for every newly established LAMP system [63]. Deviations of merely 2–3 °C can severely compromise amplification performance, manifesting as drastically reduced reaction efficiency, suppressed target yield, or rampant non-specific primer-binding and spurious amplification products [64]. Under suboptimal low-temperature regimes, faint target bands may be observable via gel electrophoresis, yet colorimetric visual discrimination becomes ambiguous and prone to subjective misclassification. While extending incubation duration up to 60 min can partially compensate for insufficient thermal energy input, this compensatory adjustment simultaneously elevates the risk of template-independent aberrant amplification and false-positive signals [65]. This extreme thermal sensitivity originates from a delicate biochemical balance: *Bst* polymerase requires sustained high strand-displacement activity while maintaining stringent primer–template binding specificity, a balance that collapses rapidly once reaction temperatures drift outside the narrow 60–65 °C operating range [66].

Under well-controlled laboratory conditions, precisely calibrated thermostatic water baths or metal blocks guarantee stable thermal conditions with marginal error typically maintained within ±1 °C, thereby eliminating temperature-induced analytical variability and enabling consistent LAMP performance [58]. However, such rigorous thermal regulation is rarely achievable in real-world field scenarios, where detection is typically conducted using portable, low-power heating modules under uncontrolled ambient conditions. Under such field constraints, the narrow ±1 °C tolerance range defined in laboratory protocols becomes practically unattainable. Studies have demonstrated that portable LAMP devices commonly exhibit temperature fluctuations of 3–5 °C during operation, which is sufficient to introduce significant inter-assay variability and undermine the reproducibility of qualitative readouts [67].

Additionally, the field practicability and translational potential of LAMP diagnostics are further restricted by the inherent stability limitations of core reaction reagents. Conventional liquid LAMP systems consist of thermosensitive biochemical components, including *Bst* strand-displacement polymerase, reverse transcriptase, dNTPs, and pH-dependent buffer substances [68]. These active constituents are prone to thermal denaturation and hydrolytic degradation under ambient or fluctuating temperature conditions, thereby requiring strict cold-chain transportation, low-temperature storage, and refrigerated operation throughout the entire detection workflow [69]. Such mandatory cold-chain dependence represents a critical practical obstacle for on-site LAMP deployment, especially in remote surveillance sites, resource-deficient regions, and emergency testing scenarios lacking stable refrigeration infrastructure. Rigorous cold-chain logistics inevitably elevate operational costs, constrain long-distance reagent distribution, shorten shelf life, and introduce assay variability induced by in-transit temperature fluctuations, collectively hindering the standardization and large-scale field popularization of LAMP point-of-care testing.

### 3.2. Results Readout Unreliability

#### 3.2.1. Endpoint Visualization Versus Real-Time Detection

Real-time LAMP relies on high-precision optical modules to continuously record full amplification-kinetic curves, enabling objective threshold definition and semi-quantitative analysis of target templates. Nevertheless, such real-time platforms demand dedicated analytical hardware, a stable power supply and professional data interpretation workflows, severely restricting their utility in resource-limited regions, remote field surveillance and rapid emergency screening scenarios. In contrast, endpoint visualization only captures optical signals upon full reaction termination, eliminating the need for continuous optical monitoring and bulky laboratory analyzers [70]. This low-cost, miniaturized workflow perfectly fulfills core on-site testing demands, including superior portability and high-throughput parallel sample screening. Accordingly, developing robust, interference-resistant endpoint readout modalities has emerged as a dominant research priority to accelerate the large-scale translational application of LAMP-based food-authenticity detection.

Despite their unparalleled merits for field deployment, conventional naked-eye endpoint readouts suffer from several interwoven, unresolved technical defects that jointly undermine analytical reproducibility: severe human subjective bias during visual discrimination, faint low-contrast color/turbidity transitions hard to distinguish by unaided eyes, and an inherent inability to biochemically separate genuine target amplicons from primer-dimer non-specific artifacts [71]. Specifically, turbidimetric LAMP relies entirely on manual visual discrimination of white magnesium pyrophosphate precipitates generated as amplification byproducts [72]. Similarly, colorimetric LAMP assays require operators to distinguish faint, nuanced hue transitions, such as subtle shifts between orange and green or pink and bright yellow [24,73]. While such label-free visual signal outputs are widely framed and promoted as a core “visualization advantage” in peer-reviewed laboratory studies, this human-dependent readout mechanism introduces critical interpretive uncertainty under real-world testing conditions [74]. In particular, weak-positive samples with marginal amplification signals often yield inconsistent qualitative results among different operators.

Such inter-operator variability generates substantial analytical uncertainty that cannot be mitigated merely by optimizing LAMP biochemistry. Controlled validation experiments demonstrate that identical post-amplification LAMP tubes assessed by three independent examiners often deliver contradictory positive/negative judgements for faint borderline signals [20]. This inconsistency does not arise from intrinsic flaws within the biochemical amplification reaction itself. Instead, it originates from variable human visual perception, a confounding human factor that cannot be eliminated by optimizing LAMP reaction formulations. Paradoxically, this naked-eye discrimination feature, widely promoted as LAMP’s flagship strength for POCT, evolves into a major barrier to standardized, objective nucleic acid detection—an essential requirement for regulatory-compliant on-site screening [75]. Beyond subjective visual bias, conventional single-step colorimetric/turbidity readouts fail to distinguish authentic target stem–loop amplicons from non-specific primer dimers and off-target amplification artifacts, both of which can produce comparable turbidity or color shifts and trigger false-positive misclassification [76]. This inability to differentiate true signals from primer-derived artifacts further exacerbates field detection unreliability, especially for multiplex LAMP systems with complex multi-primer combinations.

#### 3.2.2. Amplicon Containment and Cross-Contamination Risks

LAMP possesses extraordinary amplification potency, capable of generating a 10^9^–10^10^-fold enrichment of target nucleic acid within 30 min or less. While this ultra-high amplification efficiency enables identification of trace adulterant residues in complex food matrices [77], it concurrently renders the assay extremely vulnerable to aerosol carryover contamination. Trace amounts of amplicon carryover from prior reactions can serve as exogenous templates for secondary off-target amplification, ultimately generating spurious false-positive readouts that distort field-surveillance results [78].

This contamination vulnerability is further exacerbated by two inherent mechanistic characteristics of isothermal amplification. Unlike conventional PCR, which includes a high-temperature denaturation step that partially degrades residual amplicon contaminants, LAMP proceeds at a constant 60–65 °C without periodic thermal dissociation, allowing contaminating DNA fragments to retain full template activity for prolonged periods in ambient testing environments [79]. Compounding this structural limitation, LAMP generates lengthy concatemeric stem–loop amplicon aggregates that form characteristic ladder-like electrophoretic profiles, whose heterogeneous repetitive sequence architecture prevents unambiguous target authentication via standard Sanger sequencing [80]. Operators instead judge positive results often based on the formation of characteristic white magnesium pyrophosphate precipitates or colorimetric shifts induced by pH-sensitive dyes (e.g., hydroxynaphthol blue or phenol red) [81]. Critically, these single-channel optical readouts lack sequence-specific discrimination capacity and cannot differentiate authentic target amplification signals from artifacts triggered by residual carryover amplicons from prior testing batches.

Contamination risk evolves from sporadic isolated incidents into systemic analytical bias under typical field testing workflows lacking spatially segregated pre-amplification and post-amplification workstations [82]. Trace amplicon-laden aerosols suspended in ambient air readily infiltrate reaction vessels during routine pipetting, tube cap opening, reagent transfer, or waste disposal operations. Large-scale field validation trials quantify that airborne amplicon residues induce false-positive signals in over 20% of no-template blank control reactions when standardized anti-contamination protocols are omitted [83]. This persistent aerosol contamination bottleneck remains a major unaddressed barrier that prevents regulatory authorities from accepting an unconfirmed standalone LAMP result as evidence for formal food-authentication decisions.

## 4. The Path Forward—Building Improved-Reliability LAMP

**Figure 2:**
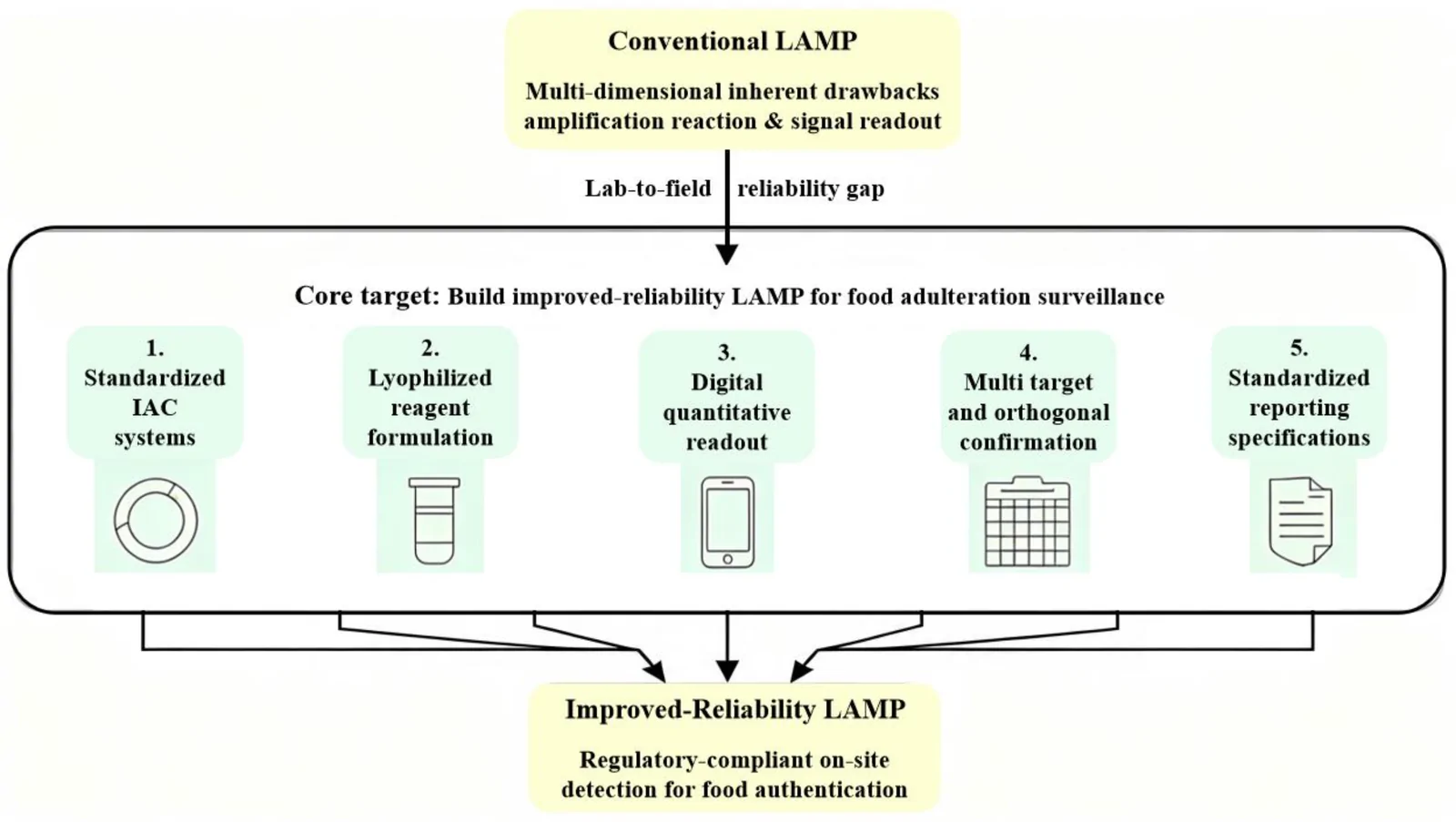
Schematic framework illustrating five coordinated multi-dimensional optimization strategies to construct improved-reliability LAMP systems for food adulteration detection, bridging the reliability gap between laboratory verification and on-site field deployment.

Conventional LAMP suffers interconnected reaction and readout defects that widen laboratory–field-performance gaps and block regulatory uptake for food adulteration surveillance. Nevertheless, highlighting these reliability deficiencies does not negate the inherent strengths and application value of LAMP as a rapid, portable, and low-cost isothermal detection platform. Rather, it emphasizes a systematic technological upgrade to transform current LAMP assays from merely functionally usable analytical tools into reliable and defensible detection approaches which, once validated for the intended purpose and coupled to a confirmatory step, can support regulatory and enforcement decisions. To bridge the lab-to-field reliability discrepancy and achieve improved reliability in on-site LAMP identification of food adulteration, this chapter proposes five coordinated, multi-dimensional optimization strategies for systematic improvement: (1) the integration of standardized internal quality control (IAC) systems, (2) the standardized fabrication and storage of lyophilized LAMP reagent formulations, (3) the replacement of subjective naked-eye interpretation with quantitative digital signal readouts, (4) the establishment of multi-scenario, multi-level assay validation frameworks, and (5) the standardized reliability-reporting specification (Figure 2). It should be noted that most of these optimization approaches represent promising technical developments derived from laboratory and pilot-scale field trials. Broad formal regulatory acceptance would still require large-scale inter-laboratory ring-testing and alignment with regional official validation guidelines.

### 4.1. Integrated Standardized Internal Amplification Control (IAC)

qPCR benefits from globally unified quality control (QC) protocols formalized by Minimum Information for Publication of Quantitative Real-Time PCR Experiments (MIQE) [84]. It incorporates endogenous references, positive/negative/no-template controls and inter-laboratory proficiency testing to eliminate systemic cross-operator/instrument error [85]. By contrast, current LAMP-based detection pipelines suffer from a pervasive and long-standing gap in standardized QC protocols, with most publications only reporting LOD and basic qualitative specificity while neglecting intra/inter-assay repeatability metrics [21,22]. This deficiency renders LAMP assays incapable of distinguishing true negative results from false-negative signals triggered by food matrix inhibitors, degraded template DNA, or complete amplification failure [43]. Notably, such systemic insufficiency in QC practice, more than any single technical limitation, forms the dominant barrier preventing unconfirmed LAMP results from being used as legally admissible evidence in regulatory enforcement [86].

As the foundational first pillar of the multi-dimensional reliability upgrading framework, standardized IAC integration is therefore an essential modification to resolve the inherent QC deficiencies of conventional LAMP [87]. Two dominant IAC formats have been proposed for LAMP detection: endogenous housekeeping gene controls and engineered exogenous competitive IACs. Endogenous IACs rely on conserved intrinsic genomic sequences within test samples. For food-authenticity testing, the mitochondrial cytochrome oxidase gene represents the most widely adopted endogenous marker, capable of verifying the integrity of DNA extraction and eliminating false negatives caused by incomplete cellular lysis [88]. In clinical RT-LAMP diagnostics for saliva or swab specimens, human 18S rRNA and RNase transcripts serve as universal endogenous references to confirm successful reverse transcription and rule out RNA degradation artifacts [89]. Nevertheless, endogenous IACs cannot normalize amplification bias stemming from inconsistent reagent dosage or matrix inhibition, restricting their application in quantitative or multiplex LAMP systems.

Exogenous competitive IACs, by contrast, offer a relatively practical strategy to achieve target detection and reaction validity assessment simultaneously within a single closed reaction tube. Synthetic artificial templates are engineered to share identical primer-binding sequences with target genes but carry unique intervening spacer fragments, enabling co-amplification within the same microenvironment without cross-interference. During isothermal incubation, IAC and target templates compete for shared limited resources, including dNTP substrates, *Bst* polymerase and primer pools. Weak or absent IAC signals indicate severe matrix inhibition or complete reaction failure, whereas robust positive IAC amplification confirms the validity of negative target results. A recent representative study established a competitive multiplex LAMP assay targeting *Listeria monocytogenes*, where the universal exogenous IAC shared identical outer/inner primer-binding sites with the pathogen target but generated a distinct melting-temperature peak (84.8 °C vs. 87.6 °C) for straightforward differentiation [90]. Subsequent validation verified that IAC integration only introduced a mild amplification time delay across all template concentrations without compromising overall assay sensitivity [86]. Despite these encouraging outcomes, the inherent challenges of achieving stable multiplex amplification remain a major technical obstacle for universal IAC embedding, slowing standardization and routine acceptance by food regulatory authorities.

Collectively, the systematic integration of diversified IAC strategies effectively fills the critical QC vacuum in traditional LAMP assays. This optimization supplies standardized calibration benchmarks to eliminate false-negative interference, enhances the stability and reproducibility of field-operated LAMP platforms, and thereby improves the method’s analytical reliability and its potential fitness for the intended screening purpose at large scale. Nevertheless, important unsolved limitations persist. Achieving well-balanced co-amplification of target and IAC templates remains technically demanding. Multiplex reaction conditions require extensive empirical tuning, and off-target cross-interference can distort signal readouts. At the maturity level, most IAC strategies exist as laboratory proof-of-concept implementations, with only a small number having undergone limited pilot field sampling trials.

### 4.2. Lyophilized Reagent Standardization for Robust Field Deployment

Traditional liquid-phase LAMP reagents impose strict low-temperature requirements for storage and transportation. Such rigid cold-chain dependency severely restricts the applicability of LAMP assays in resource-limited regions, rural markets, and unconditioned field-surveillance scenarios, where continuous low-temperature preservation cannot be guaranteed [91]. Lyophilization (vacuum freeze-drying) has emerged as a transformative, mature engineering solution to eliminate these environmental and logistical bottlenecks. By removing aqueous solvent via low-temperature sublimation, fully formulated LAMP reagents are condensed into stable dry beads. This format drastically slows enzyme denaturation, spontaneous primer dimerization, and overall reagent inactivation under ambient temperature storage conditions [92].

Lyophilization itself is not a novel concept for molecular diagnostics. Nevertheless, iterative advances in cryoprotectant screening, thermostable polymerase engineering, and large-scale uniform manufacturing over the past two years have advanced lyophilized LAMP from proof-of-concept lab prototypes to quantifiable, field-tolerant commercial diagnostic products with validated thermal stability benchmarks [93]. For pathogen detection, colorimetric lyophilized LAMP formulations incorporating neutral red and eriochrome black T dual indicators have been developed for *Streptococcus suis* screening, delivering consistent naked-eye readable signals while effectively suppressing background pH interference [69]. Broad validation across multiple pathogen detection systems further confirms that optimized lyophilized LAMP reagents can retain full biological functionality at room temperature (20–25 °C) for shelf lives of at least 28 days, demonstrating robust ambient stability for routine field surveillance [68]. Formulation-focused research has further refined cryoprotectant compound ratios, verifying that a composite of 10% trehalose combined with 2.5% dextran elevates the glass transition temperature of dry reagent matrices, mitigating physical structural collapse and gradual enzyme activity decay during prolonged room-temperature incubation [94].

Beyond cryoprotectant formula optimization, precise optimization of lyophilization processing parameters has become a pivotal research focus to further enhance bead morphology and maximize recovered enzyme activity after rehydration. Conventional single-stage freeze-drying workflow is prone to trigger severe volumetric shrinkage, porous cracking, and partial activity loss of embedded polymerases due to excessively rapid sublimation rates [95]. Emerging gradient freeze-drying protocols address these drawbacks by implementing segmented pre-freezing, primary drying and secondary drying stages [96]. Comparative trials indicate that staged lyophilization workflows improve post-rehydration Bst polymerase activity retention and lower bead fragmentation risk during industrial packaging compared with conventional single-step freeze-drying. Additional process optimization confirms that controlled slow filling of reaction cocktails into deep-well plates, combined with pre-vacuum degassing before freezing, effectively eliminates microbubble defects inside dry beads that would otherwise disrupt uniform rehydration and introduce inter-assay signal deviation [97].

The enhanced stability achieved by modern lyophilized LAMP systems is fundamentally supported by iterative protein engineering of *Bst* DNA polymerase variants. Wild-type *Bst*-LF polymerase has undergone successive rounds of rational site-directed mutagenesis and deep learning-assisted high-throughput screening, yielding a collection of optimized enzymes [98]. This panel encompasses laboratory-engineered variants including Br512, Mut235 and SC5678, alongside commercial iterations (*Bst* 2.0, *Bst* 3.0, and the newer *Bst* 4.0/5.0 series). Each successive generation exhibits incremental improvements in thermal endurance, amplification kinetics, salt tolerance, and innate resistance to food-derived matrix inhibitors [66]. When pre-complexed with thermolabile uracil-DNA glycosylase inside lyophilized beads, this engineered polymerase enables automatic enzymatic degradation of residual dUTP-containing amplicon contaminants prior to isothermal incubation, substantially alleviating the aerosol cross-contamination vulnerability that plagues conventional liquid LAMP workflows [99]. These enzyme engineering breakthroughs markedly improve the residual enzymatic activity retained post-lyophilization and after long-term ambient storage, further amplifying the inherent stability advantages of dry-bead reagent architectures.

Complementary to polymerase modification and lyophilization process upgrades, pre-integrated lyophilized microbead strips simplify cumbersome field workflows and reduce human-derived measurement bias. Commercially available 8-reaction strips preloaded with single-dose lyophilized LAMP beads achieve complete, rapid rehydration immediately upon addition of sample lysate. After rehydration, amplification efficiency and limit-of-detection are fully comparable to freshly prepared liquid reaction mixtures. This standardized “load-and-run” testing workflow minimizes repeated manual pipetting, lowers risks of accidental cross-contamination, and unifies operating protocols for untrained grassroots inspectors across diverse field sampling scenarios.

Overall, lyophilized reagent platforms optimized via cryoprotectant formulation and segmented freeze-drying processes simplify hands-on operational steps and minimize pipetting-associated variability, which helps bring the performance obtained by non-specialist field operators closer to results obtained under strictly controlled laboratory conditions. This represents a practical, scalable engineering solution that translates robust laboratory LAMP performance into more reproducible field outputs for routine food-safety and adulteration surveillance. However, this technology does not fully eliminate stability-related risks. Key unsolved limitations include inherent batch-to-batch performance variation arising from freeze-drying parameter drift, high production costs for small-batch academic laboratories, and variable long-term storage performance across different reagent compositions. Industrial-scale quality-control benchmarks for food-testing-oriented lyophilized LAMP reagents are also still not harmonized.

### 4.3. Digital Readout Replaces Subjective Naked-Eye Visual Interpretation

**Figure 3:**
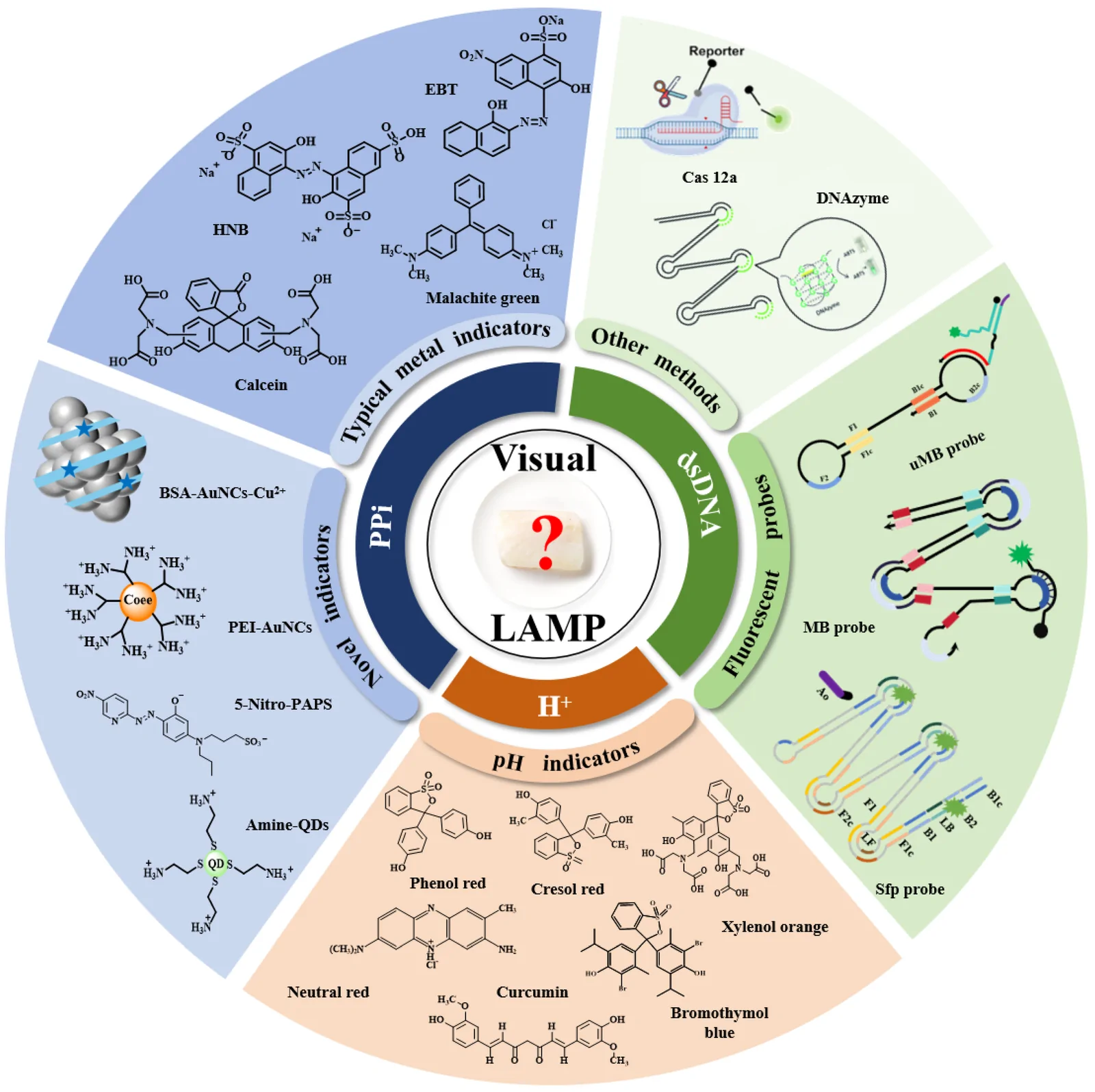
Schematic overview of diverse visual signal output strategies for LAMP assays. Visual detection mechanisms are classified according to the sensing targets generated during LAMP, including pyrophosphate (PPi)-responsive metal indicators, pH-responsive colorimetric dyes, novel nanomaterial-based indicators, double-stranded DNA (dsDNA)-binding fluorescent probes, and alternative sensing approaches relying on CRISPR-Cas and DNAzyme biosystems. Multiple categories of colorimetric/visual reporter systems have been developed for LAMP detection. (1) Typical metal ion indicators (e.g., calcein [72], HNB [100], EBT [101], malachite green [102]): these chelating dyes compete with PPi for metal cations and produce distinct color changes. (2) Novel nanomaterial-based indicators, including BSA-AuNCs-Cu^2+^ [103], PEI-AuNCs [104], Amine-QDs, and 5-Nitro-PAPS [105], rely on nanocluster/quantum dot optical responses triggered by PPi. (3) pH indicators (phenol red [102], cresol red [106], neutral red [107], xylenol orange [108], bromothymol blue [109]): monitor pH decline induced by continuous proton release throughout LAMP. (4) Fluorescent probes targeting dsDNA, including molecular beacon (MB [76], uMB [110]), Sfp probes [77] and related fluorescent hairpin constructs, emit fluorescence upon binding newly synthesized amplicons. (5) Other emerging biosensing methods integrate LAMP with Cas12a CRISPR trans-cleavage [31] and DNAzyme catalytic reporters [58] to achieve enhanced detection specificity.

A third pivotal strategy to mitigate the field reliability limitations of LAMP assays lies in the paradigm shift from subjective naked-eye visual discrimination to standardized digital quantitative readout systems (Figure 3). Over the past years, technological breakthroughs can be summarized as novel indicator chromogenic chemistry, dual-wavelength ratiometric signal amplification, sequence-specific fluorescent probing, and lightweight on-device AI analytics.

#### 4.3.1. Novel High-Contrast Indicator for Unambiguous Colorimetric Digitization

Colorimetric LAMP indicators are mechanistically divided into two major categories: magnesium ion-responsive metallochromic indicators and hydrogen ion-responsive pH chromogens, based on the chemical species they sense during amplification [81]. Magnesium ion-responsive indicators rely on competitive binding equilibrium between free Mg^2+^ and inorganic pyrophosphate (PPi) generated during LAMP. PPi precipitates Mg^2+^ to form magnesium pyrophosphate, dissociating dye–Mg^2+^ complexes and triggering color shifts. The gold-standard representative is hydroxynaphthol blue (HNB), which gradually changes from violet (negative) to pale sky blue (positive) [107]. Other conventional alternatives include eriochrome black T (EBT, purple → blue upon Mg^2+^ depletion) and xylenol orange (red → yellow) [101]. Recently, PPi-responsive nanomaterials also play a pivotal role in the development of biosensing technologies for visual LAMP, such as BSA-AuNCs-Cu^2+^ [103], PEI-AuNCs [104], and Amine-QDs and 5-Nitro-PAPS [105]. Further expanding the use of these indicators requires addressing the issue of slow, low-saturation chromatic transitions, generating broad, ambiguous intermediate color zones that readily induce inconsistent visual judgements under variable field lighting [73].

Hydrogen ion-responsive pH indicators detect protons continuously released during DNA strand synthesis, which drives a predictable bulk pH decline within the LAMP system from approximately pH 9.0 down to pH 6.5. Classical pH reporters include phenol red (red → yellow) [102] and cresol red (orange → fuchsia) [106], both widely adopted in commercial LAMP master mixes, yet their gradual color transitions still generate borderline ambiguous signals. Recently, bromothymol blue (BTB) has been shown to exhibit a more pronounced color change and greater magnitude of data variation compared with other existing indicators, with yellow indicating positive results and blue indicating negative ones [111]. Additionally, Lamalee et al. (2024) developed a one-step colorimetric LAMP assay incorporating xylenol orange for the early detection of both total *Vibrio parahaemolyticus* and specifically pathogenic *V. parahaemolyticus* [108]. Even so, similar to magnesium-sensing dyes, conventional single pH indicators lack intrinsic sequence specificity, as non-specific amplification also generates protons and induces identical color changes.

#### 4.3.2. Dual-Color Signal Enhancement to Suppress Background Interference

A series of recent studies have established integrated visual detection platforms based on mixed dual-color dye cocktails, which drastically improve naked-eye discrimination via two distinct chromogenic/fluorescent signals. All these systems enable pre-dye loading before amplification and closed-tube reactions, which substantially mitigate aerosol-derived cross-contamination risks generated after amplification by avoiding post-amplification tube-opening operations. They are also compatible with both standard tube-based assays and microfluidic digital chips. According to dye combinations and signal output modes, these dual-color strategies can be categorized into three major technical branches.

The first class consists of dual-fluorescence cocktails combining HNB with DNA intercalating dyes. The canonical HNB–SYBR Green formulation reported by Ding et al. (2015) relies on two countervailing fluorescent signals [112]: HNB emits 610 nm red fluorescence that fades as amplification consumes free Mg^2+^, while dsDNA-bound SYBR Green I produces intense 540 nm green luminescence. Distinct orange-red negative and yellow-green positive color contrasts are readily discernible without predefined cutoff values, with optimized HNB concentrations tailored for tube-based (420 μM) and self-priming compartmentalization (SPC) microfluidic formats (150–240 μM). This platform enables digital quantification of Vibrio parahemolyticus and hepatitis B virus. Substituting SYBR Green I with SYTO 9 effectively offsets its inhibition of the reaction, achieving a detection limit of 1 fg for *Salmo salar* authentication [100].

The second category is the HNB–cresol red (CR) colorimetric system that requires no external excitation light [109]. CR undergoes a magenta-to-yellow shift driven by amplification-generated protons, whereas HNB turns violet-to-sky-blue upon Mg^2+^ depletion. The superimposed two color-transition pathways generate striking hue divergence: negatives appear purple, while positives turn bright green under ambient light, with an optimal cocktail ratio of 125 μM CR plus 50 μM HNB. This low-cost strategy exhibits comparable sensitivity to real-time fluorescent LAMP for screening nut-derived food allergens.

While exhibiting strong anti-interference against aquatic and plant food substrates, all above mixed-dye dual-color systems possess an inherent drawback: they solely report bulk amplification events without conferring target-sequence specificity. To address this deficiency, a third category of probe-integrated composite dual-color systems has been devised, which endow naked-eye readout with sequence-specific recognition capability. A representative strategy couples HNB with FAM-labeled self-quenching fluorogenic primers and complementary quenching oligonucleotides [77]. This assay delivers green signals for target positives and yellow-red luminescence for negatives within 30 min, with a limit of 1 pg rainbow trout DNA and robust resistance to primer-dimer-derived false positives.

#### 4.3.3. Fluorescence-Based Readout for Improved Sequence Specificity

Diverse single- and dual-label fluorescent probe-based LAMP platforms have been developed for sequence-specific closed-tube visual detection. These platforms generate fluorescent signals only upon specific target hybridization and thus eliminate non-specific interference. Dual-label probe systems, including assimilating probes, DARQ, TaqMan-LAMP and universal molecular beacon (uMB)-LAMP, rely on FRET-based fluorophore-quencher separation triggered by amplification or enzymatic cleavage [113]. Among them, the newly proposed uMB paired with a stem–loop primer complex achieves universal applicability by only replacing partial target-recognition sequences when switching analytes, significantly cutting probe design costs [110]. Single-label probe technologies such as G-quenched Q-probes and the Ao-assisted self-quenching fluorogenic primer (Sfp-Ao) utilize intrinsic guanine fluorescence quenching or complementary oligonucleotide background suppression [77]. These approaches feature simpler synthesis workflows and lower associated expenses. Notably, the Sfp-Ao composite strategy integrated with HNB dual dyes perfectly combines the sequence specificity of fluorescent probes and the high naked-eye color contrast of mixed dyes, producing distinct green positive and orange-yellow negative signals within 30 min with a detection limit of 1 pg DNA [77].

Compared with pure mixed-dye LAMP, probe-based LAMP delivers superior target specificity, single-nucleotide variant discrimination capacity and multiplex detection performance, albeit with slightly higher reagent costs and stricter cold-storage requirements for fluorescent probes [114]. These probe-mediated LAMP assays have been widely applied in food-authenticity identification, food-borne pathogen screening and clinical infectious virus detection, and can be matched with microfluidic chips for digital absolute quantification. Nevertheless, current fluorescent probe LAMP techniques still face challenges including poor thermal stability of modified probes, limited multiplex throughput and matrix-induced background noise [115]. To tackle these limitations, follow-up research will focus on three directions: constructing integrated probe-dual dye lyophilized one-pot reagents, optimizing universal modular probe frameworks, and matching miniaturized portable fluorescence readers to expand the applicability of this technique in low-resource field testing.

#### 4.3.4. AI-Assisted Smartphone Digital Platform for Endpoint Quantification and Automated Judgment

While colorimetric LAMP assays deliver intuitive visual contrast between positive and negative amplification outcomes, naked-eye judgment remains vulnerable to subjective human color bias and inconsistent ambient light interference [24]. To address this limitation, portable smartphone imaging hardware combined with lightweight edge deep learning (DL) algorithms enables fully automated signal quantification, upgrading LAMP readout from binary qualitative judgment to absolute nucleic acid quantitation [116]. Convolutional neural networks (CNNs), the mainstream backbone for biosensing image analysis, exhibit exceptional capability to extract subtle chromatic and fluorescent pixel features. Well-trained CNN pipelines can simultaneously perform food ingredient authentication, hazardous contaminant screening, and geographic origin tracing with superior analytical precision. An early study constructed an automated image acquisition workflow coupled with preliminary AI preprocessing modules, which shortened the total assay turnaround time and mitigated the subjective deviations inherent to manual color scoring of LAMP mixtures [117]. Subsequent research further optimized CNN-based pixel segmentation algorithms to accelerate batch testing workflows, enabling consistent, unbiased result reading for large-scale food safety sampling campaigns [118].

Driven by the widespread adoption of mobile sensing hardware, technical breakthroughs have shifted LAMP detection toward decentralized point-of-care (POC) paradigms. Modern smartphones, equipped with high-sensitivity CMOS cameras and embedded edge computing chips, can quantify color signals under standardized RGB or CIELAB uniform color spaces, combining the advantages of low procurement cost, outstanding portability, and simplified field operation [119]. Beyond standalone mobile terminals, the synergy of DL algorithms and Internet of Things (IoT) sensors unlocks end-to-end intelligent detection pipelines, as exemplified by Hilab Molecular’s integrated POC platform [120]. This IoT-AI integrated system autonomously captures reaction imagery, executes on-device neural network inference, and synchronizes encrypted digital records to cloud servers, supporting real-time remote supervision of food adulteration and microbial contamination incidents.

Collectively, the multi-dimensional digital readout system built on AI-empowered smartphone platforms resolves the core limitations of traditional naked-eye visual interpretation. This integrated digital readout framework represents an indispensable technical upgrade to address the “readout unreliability” dimension of LAMP’s inherent detection bias, drastically unifying result consistency across geographically dispersed field inspection sites and generating archivable, traceable digital records with potential to support regulatory assessment for routine food-authenticity governance [36]. Nevertheless, current portable AI-LAMP systems still face constraints, including limited on-chip computing power, insufficient multiplex detection capacity, and model generalization bias toward rare food matrices. Future development trends will focus on lightweight multi-modal fusion models, ultra-low-power portable optical modules, 5G-cloud collaborative data management, and blockchain-based detection record storage. Such technical upgrades will promote the large-scale deployment of intelligent LAMP testing in scenarios such as food-authenticity supervision, pathogen emergency screening, and grassroots on-site inspection.

### 4.4. Multi-Target and Orthogonal Confirmation Strategies

As the fourth core pillar of our five-dimensional upgrading framework to mitigate LAMP’s inherent unreliability, the multi-strategy consensus validation framework mitigates pervasive false-positive risks stemming from conventional single-target LAMP detection, a major source of unreliable readouts summarized in the preceding schematic diagram. Traditional single-gene LAMP assays rely solely on one nucleic acid biomarker for qualitative judgment, forming a high-risk “single-evidence” diagnostic paradigm [121]. Such assay set-ups can generate misleading signals. These signals arise from non-specific primer cross-amplification, aerosol amplicon carryover contamination, and heterogeneous matrix interference encountered in real-food samples. Over the past three years, duplex collaborative multi-target detection has matured and achieved practical field deployment in food-authenticity surveillance. The most prevalent implementation enables parallel screening of two evolutionarily independent genetic markers, exemplified by paired species-specific virulence genes for food-borne pathogens such as *Listeria* spp. and *Listeria monocytogenes* [122]. Comparative field validation versus single-target LAMP confirms that duplex consensus calling criteria can substantially lower field false-positive rates and improve resistance to inhibitory substances present in complex meat, aquatic and plant matrices. Building upon duplex detection, continuous technical advances have extended multiplex isothermal amplification to standardized triplex synchronous validation [123]. Its key innovations lie in large-scale primer-probe screening databases and refined buffer tuning, tackling two dominant bottlenecks limiting multiplex LAMP performance: imbalanced amplification kinetics across distinct targets and inherent cross-reactivity between discrete primer sets. Standardized melting-temperature calibration combined with orthogonal sequence design effectively suppresses uneven amplicon yields and inter-primer interference. This renders triplex and higher-order parallel detection sufficiently stable and reproducible to serve, prospectively, as a screening tool within regulatory field-surveillance programs.

Beyond multi-target parallel amplification, integrated CRISPR/Cas effector systems introduce a next-generation, high-specificity orthogonal verification module to complement multi-target LAMP consensus judgment, constructing a dual-layer molecular validation paradigm to eliminate ambiguous readouts [124]. Cas12a and Cas13a possess intrinsically orthogonal substrate cleavage preferences, supporting independent parallel signal decoding without mutual interference: Cas12a specifically recognizes and cleaves double-stranded DNA LAMP amplicons, whereas Cas13a targets T7-transcribed single-stranded RNA transcripts generated from modified LAMP primer sets. This characteristic enables spectrally separated fluorescent or lateral flow signals for dual-channel consensus confirmation. When synergistically combined with microfluidic chips, CRISPR-enhanced multiplex LAMP systems reduce sample consumption to microliter scale, support battery-powered constant-temperature incubation without laboratory thermocyclers, and facilitate closed-tube one-pot amplification-cleavage workflows that drastically cut aerosol contamination risks [71]. These miniaturized all-in-one platforms have undergone systematic field validation at port quarantine stations, grassroots farm sampling sites and mobile testing facilities, demonstrating robust portability and favorable environmental tolerance under uncontrolled ambient temperature conditions. To date, the main unsolved drawbacks are increased assay complexity, elevated reagent cost, and amplification-kinetics imbalance among parallel target markers. Multiplexed systems demand heavy pre-experimental optimization to avoid signal suppression. Limited real-food pilot trials have been reported, while standardized protocols for accredited testing workflows are still absent.

### 4.5. Standardized Reliability-Reporting Specification

The final and most transformative optimization dimension lies in establishing standardized reliability evaluation and reporting specifications. This framework places reliability metrics on an equal statutory and academic footing alongside conventionally prioritized indicators including detection sensitivity and amplification speed. This shift represents one of the most underappreciated yet far-reaching directions guiding iterative LAMP improvements. It fundamentally corrects the enduring research bias that overemphasizes analytical sensitivity while overlooking assay robustness under field conditions.

In recent years, Chinese national and industrial standards have gradually introduced mandatory requirements for quality control documentation, laying an institutional foundation for standardized reliability assessment of isothermal amplification assays. The implementation rules of the exported food inspection standard series (SN/T 2754) explicitly mandate parallel deployment of positive controls, negative controls and internal amplification controls throughout complete LAMP workflows [125]. This requirement curbs arbitrary, fragmented and inconsistent QC arrangements widely observed in early laboratory studies, which frequently fail to validate reaction validity and produce questionable experimental data. Furthermore, the national technical specification for quality evaluation of nucleic acid amplification kits (GB/T 37871-2019) defines quantifiable benchmarks for assay repeatability [126]. It adopts intra-assay coefficients of variation (CV) to assess within-batch repeatability and batch-to-batch consistency, and relies on multi-site collaborative trials to set inter-laboratory reproducibility thresholds, delivering unified quantitative criteria for evaluating LAMP operational robustness in complex food matrices. Additionally, the port inspection standard series (SN/T 3306) further optimizes full-chain standardized specifications covering the entire detection pipeline, including field sample collection, pretreatment purification, constant-temperature amplification operation, and endpoint result judgment, realizing systematic standardization and normative constraint for every procedural link of LAMP detection [127].

Relevant international resources include ISO/TS 16393 for validation of qualitative molecular-detection methods, and EU official-control paradigms mandating that presumptive screening results require orthogonal confirmatory testing prior to enforcement action [40]. Comprehensive internationally agreed performance benchmarks, inter-laboratory ring-trial datasets across diverse food-adulteration matrices, and formal acceptance criteria differentiating screening versus confirmatory LAMP testing remain largely under development. Validation must go beyond reporting only limit-of-detection and specificity measured under ideal laboratory buffer-spiked conditions. Fitness-for-intended-purpose assessment should incorporate matrix-matched real-food samples, inter-operator variability assessment, and pilot-scale field-performance data.

Driven by advancing regulatory frameworks, a cross-industry consensus on standardized result reporting is rapidly taking shape within the molecular diagnostics community. Reliability and reproducibility indicators are no longer regarded as marginal supplementary descriptions, but are required to be independently quantified, analyzed, and reported equivalently with core indicators such as detection sensitivity, specificity, and LOD in all methodological validation studies and commercial product certification documents [128]. Leading global suppliers of LAMP reagents, including Eiken Chemical, New England Biolabs, and Nippon Gene, have responded proactively to this standardization trend. Where required by regulatory authorities, these manufacturers integrate batch-to-batch stability statistics, multi-environment tolerance profiling, and cross-laboratory collaborative validation results into official product registration dossiers and supporting peer-reviewed studies. This industrial practice promotes the comprehensive transformation of LAMP reliability evaluation from traditional qualitative empirical judgment to modern standardized quantitative disclosure, effectively bridging the application gap between laboratory-based technical optimization and regulatory-compliant industrial promotion.

Standardized reliability-reporting specifications establish unified metrics for evaluating repeatability, reproducibility and matrix tolerance, filling a long-standing research gap where assay performance was historically dominated by reporting only limits-of-detection and amplification speed. Notwithstanding these benefits, substantial practical barriers remain. Existing regional nucleic acid kit standards (SN/T, GB/T) are generic documents and are not fully tailored for LAMP-specific food-authentication workflows. Moreover, globally harmonized international validation benchmarks built upon frameworks such as ISO/TS 16393:2019 are still lacking. Inter-laboratory ring-trial datasets for food-adulteration matrices with agreed reporting criteria distinguishing screening versus confirmatory testing are sparse, and no harmonized criteria yet exist for the fitness-for-intended-purpose evaluation that must precede regulatory acceptance. In summary, this five-dimensional optimization framework, integrating reaction calibration, lyophilized reagent engineering, digital signal readout, multi-strategy consensus verification and normative performance evaluation, addresses key layered inherent limitations of conventional LAMP technology. It unifies performance evaluation logic and credibility judgment criteria for on-site testing, delivering robust technical and normative support for broader regulatory consideration of LAMP in food-authenticity screening, food-borne pathogen surveillance and grassroots food safety supervision.

## 5. Next Decade of LAMP: From Analytical Performance to Operational Trust

Over the past two decades, LAMP has emerged as one of the most powerful molecular tools for detecting food adulteration [9]. The analytical advantages have been well documented and collectively distinguished it from most alternative isothermal amplification methods in terms of practical field deployment. However, as the technology matures, it is important to recognize that amplification speed and detection sensitivity are no longer the main barriers limiting its broader adoption. These performance metrics have largely been improved to levels that meet or exceed the analytical-performance expectations of most regulatory and industrial applications [50]. Instead, the factor that will shape the future trajectory of LAMP is operational reliability. Specifically, this encompasses the reproducibility and robustness of test outcomes across operators, laboratories, heterogeneous food matrices, and formal regulatory environments. In practice, LAMP assays remain susceptible to multiple well-characterized sources of variability, such as narrow thermal tolerance, aerosol cross-contamination, inherent false-positive and false-negative risks, subjective naked-eye signal interpretation, and the absence of harmonized quality-control frameworks [54]. Such limitations are manageable under strictly controlled laboratory research conditions. They become critical vulnerabilities, however, when identical assays must produce consistent, defensible results within regulatory inspection facilities, industrial quality-control laboratories, or formal legal proceedings.

To address these challenges, the five aforementioned optimization strategies constitute a mutually nested, synergistic, and systematic improvement ecosystem. The integration of embedded internal quality control systems is inherently compatible with lyophilized reagent standardization, enabling one-tube embedded QC implementation in ready-to-use dry beads. Digital quantitative readout systems provide objective, unified, and traceable signal criteria for multi-target consensus validation, eliminating subjective interpretation bias in high-plex detection. Most critically, standardized reliability-reporting specifications serve as the top-level normative constraint. They enforce consistent implementation of the preceding four technical optimizations and guide every technical upgrade toward verifiable, repeatable, and field-performance fit for the defined intended purpose. At present, partial core technical components, scattered industrial proof-of-concept verification cases, and region-specific statistical evaluation and normative documents for reliable LAMP field deployment are available. Nevertheless, substantial regional variability exists: comprehensive cross-jurisdictional industrial validation datasets and globally harmonized national standard systems for food-oriented LAMP testing are still under ongoing development and are not universally established across all regulatory authorities. The remaining bottleneck is therefore not purely a technical deficiency. It also requires a paradigm shift within the research and industrial community: redefining reliability and operational robustness as core design principles equal to speed and sensitivity, and working toward transforming LAMP from a highly sensitive laboratory screening tool into a more trustworthy, standardized analytical instrument for food authentication and public health surveillance.

In summary, the next decade of LAMP development will not be defined by how fast the technology can detect a target, nor by how low a concentration it can identify. Instead, progress will be defined by a fundamental criterion: whether any stakeholder—regulator, industry, or consumer—can rely on a LAMP-derived result without reservation, and whether that result can withstand the procedural rigor demanded by formal validation frameworks and adversarial legal proceedings. The central question has therefore shifted decisively from “How sensitively can LAMP detect targets?” to “How consistently trustworthy are LAMP results?” Addressing this question will require not only continued optimization of assay chemistry, but also the establishment of standardized validation protocols, inter-laboratory proficiency testing schemes, and regulatory acceptance criteria that recognize LAMP not merely as a laboratory research tool, but as a promising preliminary screening analytical technology. It still requires purpose-specific formal fitness-for-intended-purpose validation, adherence to screening-plus-confirmation workflows, and alignment with international official-control requirements before it can be considered for regulatory-relevant food-authenticity surveillance. Importantly, this process confers screening utility rather than confirmatory status. Elevated analytical sensitivity and technical robustness do not by themselves make a LAMP assay suitable for regulatory or legal decision-making, which additionally requires purpose-specific validation, confirmation of presumptive positives by an independent reference method, and acceptance by the competent authority.

## Figures and Tables

**Table 1:** Representative nucleic acid-based amplification assays for food species authentication.

Application Scenario	NA Method	Integrated Technology	Highlights	Reference
Salmon adulteration detection	RAA of the *myoglobin* nuclear gene	(1) CRISPR-Cas12b for sequence-specificity. (2) Microfluidic chip for lab-free and on-site detection. (3) LFA using a multifunctional Pt3Sn@MGO nanocomposite for signal output.	(1) Detection limit of 0.153%, 0.092%, and 0.007% (*w*/*w*) in colorimetric, photothermal, and fluorescent modes, respectively.	[12]
Multi-species meat authentication	RPA of the 12S rRNA gene	(1) CRISPR/Cas12a for sequence-specificity.	(1) Detection limit as low as 0.05% (*w*/*w*) in binary mixtures. (2) Rapid workflow (~40 min) with a low material cost of $4.17 per test. (3) Universal RPA primers enable parallel single-target identification of 11 meat species.	[13]
Milk powders authentication	RPA of several mitochondrial genes	(1) Lateral flow strip (LFS). (2) Real-time fluorescence.	(1) LOD reaches 0.5% for camel, 0.1% for goat, 1.0% for donkey and 0.1~0.5% for cow (in milk powder mixtures). (2) Approximately 30~35 min from sample to result.	[14]
Duck, donkey, and sheep/goat authentication	ERA of the species-specific single-copy nuclear genes	(1) Lateral flow strip (LFS).	(1) Detected as few as 0.016 ng genomic DNA per reaction within 30 min.	[15]
Mithun (*Bos frontalis*) meat authentication	PSR of the D-loop gene	(1) Visual result interpretation using SYBR Green I dye.	(1) Enabling detection of target DNA up to 10 fg.	[16]
Detection of pufferfish	PCR of the COI gene	(1) CRISPR/Cas12a for sequence-specificity. (2) ECL biosensor using NiCo_2_O_4_ NCs@Au-ABEI as nanoemitters.	(1) Detection limit of 0.1% (*w*/*w*).	[17]
Detection of pork adulteration	LAMP of the CYTB gene	(1) CRISPR/LbCas12a for sequence-specificity.	(1) Detection limits of 0.05% (*w*/*w*) for binary mixtures, and 50 pg/μL for DNA limit.	[18]
Silkworm detection	LAMP of the COI gene	(1) CRISPR/Cas12a for sequence-specificity.	(1) Detection limits of 0.1 pg in silkworm genomic DNA and 0.01% (*w*/*w*) in the spiked sample.	[19]
Detection of pork adulteration	LAMP of the D-loop gene	(1) HNB and calcein for visual detection. (2) A self-designed, portable device to achieve both amplification and dual-channel detection.	(1) Detect pork as low as 0.1% in adulterated samples. (2) This device is cost-effective, priced at only $54.65.	[20]
Three cockle species authentication	LAMP of the COI gene	(1) Agarose gel. (2) Real-time fluorescence.	(1) Detection limit demonstrated up to 0.0001 ng genomic DNA.	[21]
Lethal mushroom authentication	LAMP of the ITS gene	(1) CRISPR/Cas12a for sequence-specificity.	(1) Visual detection limit achieved as low as 1 fg/μL of DNA or 0.01% mushroom admixture.	[7]
On-site identification of sturgeon	LAMP of the CYTB gene	(1) Dual-mode LAMP integrates SYTO-9 fluorescence and HNB colorimetry in a closed-tube format.	(1) Detects 0.1 pg of Acipenser sinensis DNA within 10 min at 64 °C.	[22]
Authentication of soybean oil	LAMP of the ITS2 gene	(1) Real-time LAMP.	(1) Sensitivity of rtLAMP for soybean DNA authentication in oil samples was 10^−3^ ng.	[3]
*Salmo salar* authentication	RPA of the CYTB gene	(1) CRISPR/Cas12a. (2) Lateral flow strip (LFS). (3) Visual inspection.	(1) Sensitive genomic DNA detection with a vLOD of 5 copies/μL. (2) Identification of 5% *w*/*w S. salar* in *O. mykiss* and *S. salar* mixtures. (3) Semi-quantitative detection in processed samples with 93.3–100% accuracy.	[23]

**NA:** nucleic acid amplification; **LAMP:** loop-mediated isothermal amplification; **RAA:** recombinase-aided amplification; **RPA:** recombinase polymerase amplification; **ERA:** enzymatic recombinase amplification; **PSR:** polymerase spiral reaction. This table summarizes published application-level performance indicators including detection limit, assay runtime and integrated hardware/readout modules. Comparative reliability analysis across PCR and different isothermal amplification modalities with respect to reaction-level and readout-level failure modes is presented in the main text, rather than within table cells, to prevent table overcrowding. A small subset of pathogen-oriented technical references are retained only to illustrate general assay-performance characteristics and are not the primary focus of this review.

## Data Availability

The data presented in this study are available on request from the corresponding authors due to privacy.
